# Anesthetic Management of a Parturient With Lipomyelomeningocele and Tethered Cord for Cesarean Section

**DOI:** 10.7759/cureus.105820

**Published:** 2026-03-25

**Authors:** Sara Matos, Marta Marques, Ana T Magalhães, Catarina Sampaio, Marta Guerra

**Affiliations:** 1 Anesthesiology, Unidade Local de Saúde Alentejo Central, Évora, PRT; 2 Anesthesiology, Unidade Local de Saúde São João, Porto, PRT

**Keywords:** cesarean section (cs), general anesthesia, lipomyelomeningocele, obstetric anesthesia and analgesia, spinal dysraphism

## Abstract

Lipomyelomeningocele (LMMC) presents significant anesthetic challenges due to spinal cord tethering and distorted anatomy, which make the gold-standard neuraxial anesthesia technically difficult and neurologically risky. We report the case of a 29-year-old primigravida with LMMC and prior neurosurgeries scheduled for an elective cesarean section. Given the risk of direct neural injury and unpredictable block spread, a planned general anesthesia (GA) was performed, and a multimodal analgesic strategy was employed. The procedure was uneventful, resulting in a healthy neonate and a mother with excellent pain control and no deviation from her neurological baseline. This case demonstrates that while neuraxial techniques are usually preferred, GA is a safe and necessary alternative for patients with complex spinal dysraphism. Success requires a thorough preoperative neurological assessment, neuroimaging review, and tailored multidisciplinary approach.

## Introduction

Neural tube defects (NTDs) are congenital malformations resulting from the failure of neural tube closure during embryogenesis. They are broadly classified as open or closed based on whether the neural tissue is exposed or covered by skin [1]. Closed NTDs (closed spinal dysraphisms) are characterized by a defect covered by skin. These include spina bifida occulta, lipomyelomeningocele, tethered cord, and split cord malformations [1-3].

Lipomyelomeningocele (LMMC) is a prototypical closed NTD, presenting as a subcutaneous lipomatous mass that extends through a spinal defect and is intimately associated with the spinal cord and meninges [3]. While these skin-covered lesions are most frequently localized to the lumbosacral region, cervical and thoracic presentations have also been documented [4]. Clinically, LMMC typically presents as a skin-covered soft tissue mass, often accompanied by cutaneous stigmata such as dimples or discoloration [3]. Neurological sequelae, including progressive motor deficits, bladder dysfunction, and pain, arise from tethered cord syndrome [3,4]. Magnetic resonance imaging (MRI) remains the gold standard for definitive diagnosis [5].

In the obstetric population, LMMC presents a significant anesthetic dilemma. While neuraxial techniques are the gold standard for cesarean sections due to maternal and fetal benefits, the presence of spinal dysraphism introduces technical and safety concerns. The anesthesiologist must weigh the risks of unpredictable block spread and potential direct neural injury against the well-known risks of general anesthesia in a pregnant patient, such as difficult airway management and aspiration [6,7].

The prevalence of neural tube defects varies geographically. In Portugal, the reported prevalence is approximately 4.2 per 10,000 births, while across European countries, the prevalence is estimated at around 9 per 10,000 births according to data from the European Surveillance of Congenital Anomalies (EUROCAT) [8,9]. Because these conditions are relatively rare, clinicians often lack frequent hands-on experience; therefore, understanding their neurological complexity and severity is paramount to identifying perioperative risks and delivering optimal anesthetic care.

## Case presentation

A 29-year-old primigravida, American Society of Anesthesiologists (ASA) physical status III, at 39 weeks and 2 days of gestation, was scheduled for an elective cesarean section due to breech presentation. The patient had a known history of lipomyelomeningocele, having undergone multiple corrective neurosurgeries during childhood. Her documented neurological sequelae included a neurogenic bladder requiring intermittent catheterization, a neurogenic bowel, and a motor deficit characterized by the inability to perform plantarflexion of the foot. Physical examination upon admission confirmed these findings.

**Figure 1:**
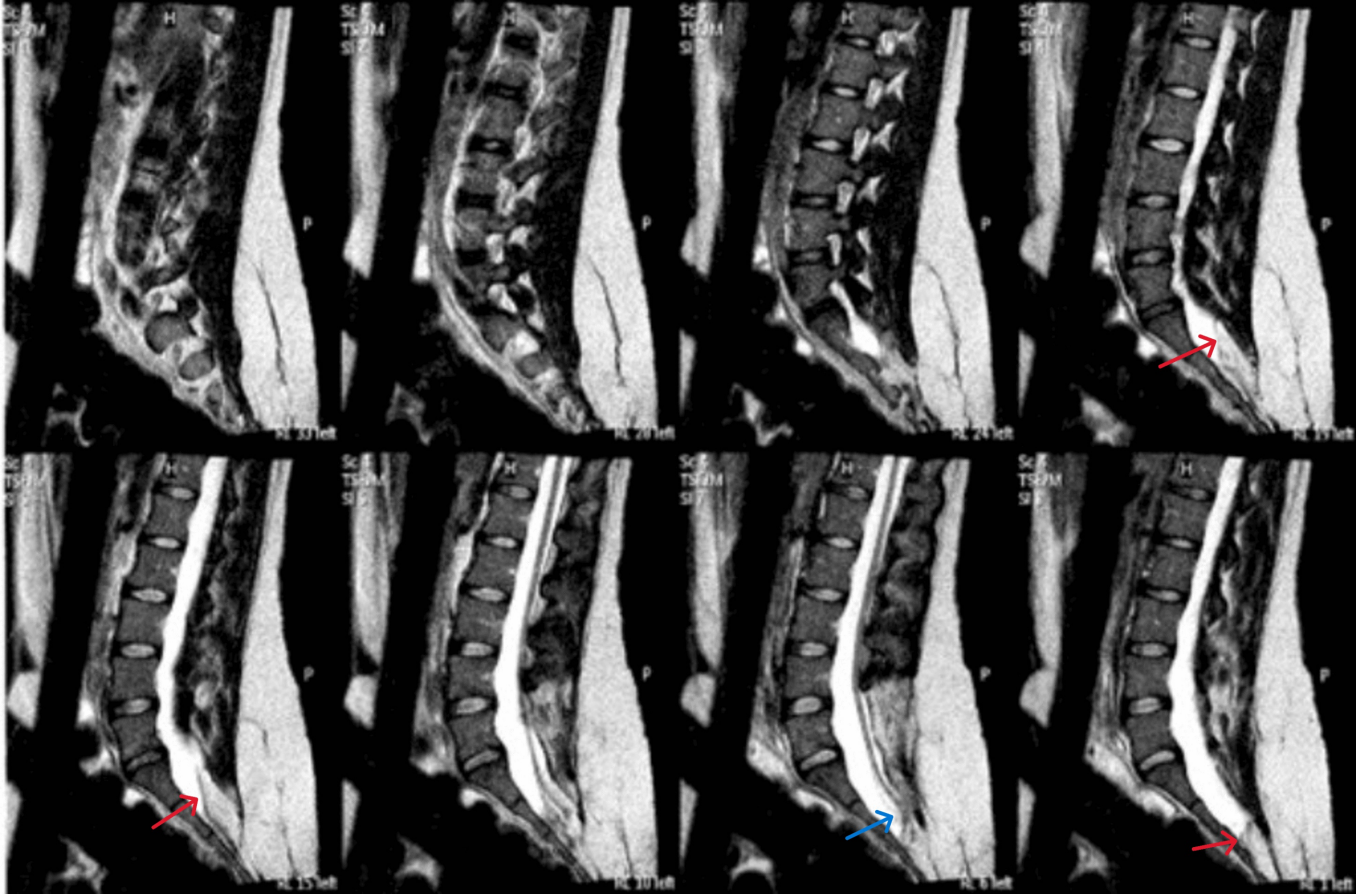
Magnetic resonance imaging (MRI) of the lumbosacral spine Sagittal T2-weighted MRI views demonstrating a complex closed spinal dysraphism consistent with lipomyelomeningocele (red arrow) and tethered cord (blue arrow) syndrome.

The pre-anesthetic evaluation included a comprehensive clinical history, airway assessment, and a detailed neurological examination to establish a baseline. Her airway examination revealed a normal neck circumference, preserved neck extension, and a thyromental distance (TMD) > 6 cm. The upper lip bite test (ULBT) was Grade 1, and the Mallampati score was 2. Review of prior magnetic resonance imaging (MRI) confirmed the presence of a persistent lumbar lipoma, low-lying cord, and a tethered cord syndrome (Figure 1).

Considering the patient’s complex spinal anatomy and the potential risks associated with neuraxial techniques, specifically the unpredictability of the spread of the neuraxial block, the efficacy, and the risk of direct neural injury, general anesthesia was selected after a thorough discussion of the risks and benefits with the patient.

Acid aspiration prophylaxis was provided with 40 mg of pantoprazole and 10 mg of metoclopramide 30 minutes prior to the procedure. The patient was positioned with a left lateral wedge to prevent aortocaval compression and was pre-oxygenated for three minutes, achieving an expired oxygen fraction (EtO2) over 90%. Standard and advanced intraoperative monitoring was established to ensure maximum safety, including pulse oximetry, capnography, five-lead ECG, and objective neuromuscular monitoring via train-of-four (TOF). Additionally, the bispectral index (BIS) and continuous EEG were utilized to titrate the depth of anesthesia accurately, minimizing the risk of intraoperative awareness while avoiding excessive exposure to anesthetic agents.

Rapid sequence induction was performed using 90 mg of lidocaine, 200 mg of propofol, and 90 mg of rocuronium. Eight mg of dexamethasone was also administered for antiemesis and pain analgesic properties. Intubation was successfully achieved on the first attempt using a size 7.0 mm endotracheal tube (ETT) and videolaryngoscopy. Correct ETT placement was confirmed by clinical assessment (bilateral chest rise and auscultation) and the presence of a persistent capnography waveform. Anesthesia was maintained with end-tidal sevoflurane 1.8% and titrated to a BIS target of 50, ensuring an adequate hypnotic state and preventing intraoperative awareness during the pre-delivery phase.

The procedure was uneventful, resulting in the delivery of a healthy neonate with Apgar scores of 7, 8, and 10 at 1, 5, and 10 minutes, respectively.

Following the delivery of a healthy neonate, sevoflurane was discontinued to mitigate the risk of dose-dependent uterine atony. Anesthesia was transitioned to a total intravenous regimen with a propofol 1% infusion at a rate of 25 mL/h (approximately 64 mcg/kg/min). To further ensure uterine tone, a bolus of 10 IU of oxytocin was administered slowly, followed by a continuous infusion of an additional 10 UI. Multimodal analgesia was optimized with 150 mcg of fentanyl, 1 g of paracetamol, 30 mg of ketorolac, 2 g of magnesium sulfate, and 4 mg of morphine. For regional postoperative pain control, a bilateral ultrasound-guided transversus abdominis plane block was performed using 40 mL of 0.375% ropivacaine.

At the end of the surgery, a neuromuscular blockade was fully reversed with 200 mg of sugammadex. Extubation was performed once the patient was fully awake, protective airway reflexes were restored, and a train-of-four (TOF) ratio > 0.9 was objectively confirmed, ensuring no residual paralysis. The patient was transferred to the post-anesthesia care unit (PACU) for close monitoring.

Following an uneventful two-hour recovery in the PACU, the patient was transferred to the obstetric ward. On the first postoperative day, a follow-up visit was conducted to screen for delayed complications and evaluate the efficacy of the multimodal analgesic regimen. This regimen consisted of scheduled intravenous paracetamol (1 g every 6 hours) and ketorolac (30 mg every 8 hours), with intravenous morphine (4 mg) reserved for breakthrough pain. The patient reported excellent pain control, with a reported visual analog scale (VAS) score of <3/10 lying at rest, likely secondary to the prolonged effect of the bilateral TAP block and the synergistic effect of the systemic analgesics. Most importantly, a focused neurological examination confirmed that the patient remained at her preoperative baseline, with no evidence of new sensory or motor deficits.

## Discussion

Due to the rare presentation of the patient with LMMC undergoing cesarean section, we wished to highlight our strategic approach to the anesthetic management in this elective setting at our hospital.

In this particular case, an elective cesarean section was required due to breech presentation. Two main anesthetic approaches were considered: neuraxial anesthesia and general anesthesia.

Generally, we avoid general anesthesia for cesarean delivery because of the associated maternal risks, such as failed airway management, aspiration, surgical site infection, venous thromboembolism, and increased postoperative complications, including greater blood loss [8]. Neuraxial anesthesia remains the gold standard for cesarean delivery [9].

The decision to proceed with general anesthesia was primarily driven by the patient's complex spinal anatomy. In cases of spinal dysraphism, such as lipomyelomeningocele with a low-lying conus medullaris, neuraxial techniques carry significant risks. These include the unpredictability of block spread and efficacy due to potentially distorted epidural or subarachnoid spaces, as well as an increased risk of direct neural injury during needle or catheter placement. Furthermore, the presence of prior corrective surgeries and scarring could lead to an incomplete or patchy block, necessitating an unplanned intraoperative conversion to general anesthesia under less-than-ideal conditions. By opting for a controlled general anesthetic from the beginning, we prioritized maternal safety and ensured a stable surgical field.

## Conclusions

Given the low incidence of lipomyelomeningocele (LMMC), clinicians may not always have immediate experience with the optimal anesthetic management for these patients. Management presents a significant clinical challenge, as it requires balancing the known risks of general anesthesia in obstetrics against the technical and neurological hazards associated with neuraxial techniques. While neuraxial anesthesia is typically preferred for cesarean sections, this case highlights that general anesthesia remains a prudent and necessary alternative when spinal anatomy is severely distorted. The presence of complex spinal dysraphism, characterized by altered anatomy, surgical scarring, and low-lying neural structures, can make neuraxial blocks unpredictable and dangerous.

Ultimately, this case underscores that a thorough preoperative neurological assessment, a detailed review of neuroimaging, and a tailored anesthetic plan, developed through shared decision-making with the patient, are essential for a successful outcome. This experience reinforces that general anesthesia is an appropriate, and sometimes safer, choice in selected patients with complex spinal dysraphism.
